# Supplementary figures and images for: A complex genetic architecture in zebrafish relatives Danio quagga and D. kyathit underlies development of stripes and spots
Source: PLoS Genet. 2021 Apr 26;17(4):e1009364. doi: 10.1371/journal.pgen.1009364 (PMC8102007; doi:10.1371/journal.pgen.1009364)

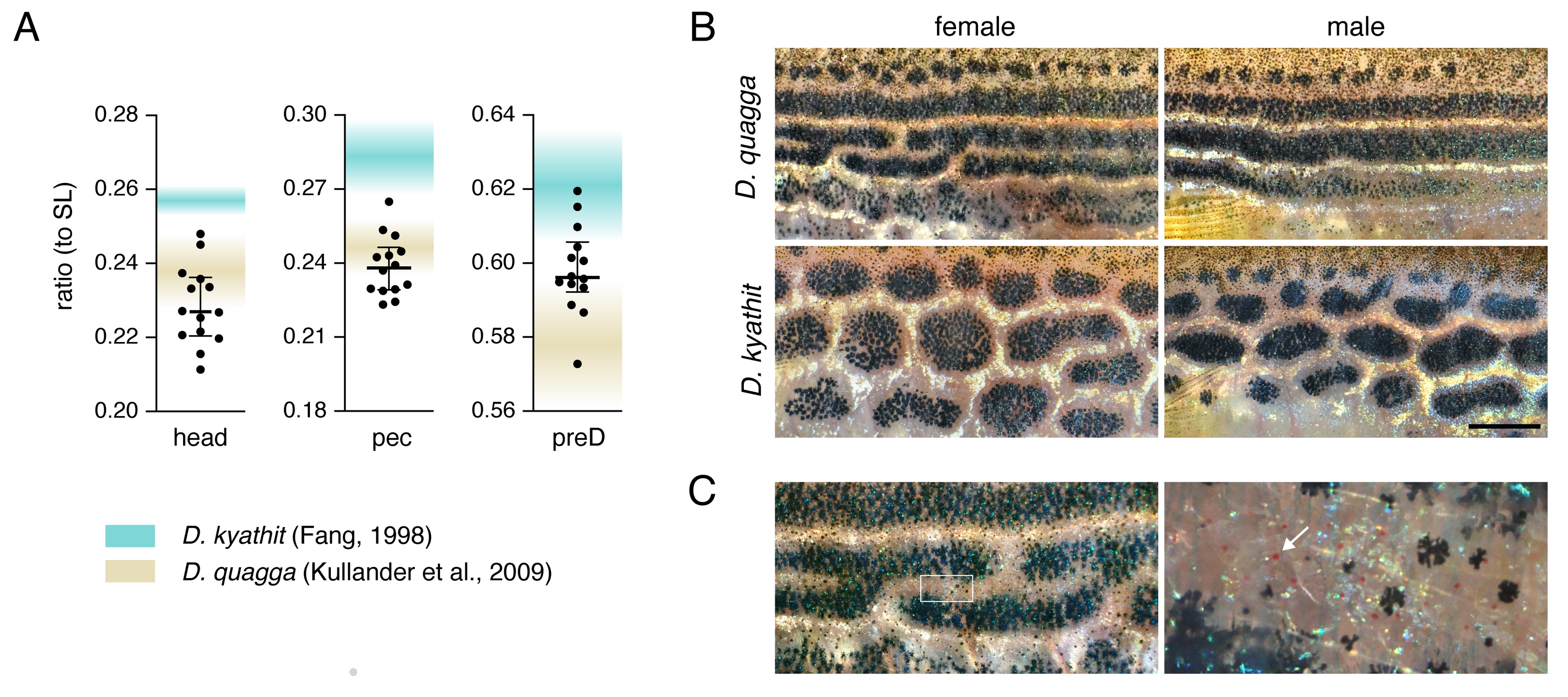

Supplement: S1 Fig — (A) Body proportions of striped fish used in this study were more similar to proportions described originally for D. quagga than D. kyathit, based on the small numbers of preserved specimens on which original species descriptions were based (n = 5 and n = 6, respectively) [47,48]. Shaded regions in plots indicate approximate proportions as inferred from published reports. Points and bars (median ± interquartile range) represent adult fish (n = 14) sampled from stocks used in this study. head, head length; pec, pectoral fin length; preD, pre-dorsal fin length. (B) In adult D. quagga, stripes are initially uniform (Fig 2) but develop fissures and reticulations after several months as fish continue to grow. These are particularly evident in deeper bodied females (upper left, ~35 mm SL). In D. kyathit, the early adult pattern is maintained during later stages. (C) Details of female D. quagga in A, illustrating stripe reticulations (left) and, in the boxed region, red erythrophores (arrow, right). Scale bar, 2 mm in B. (TIF) [file pgen.1009364.s001.tif]

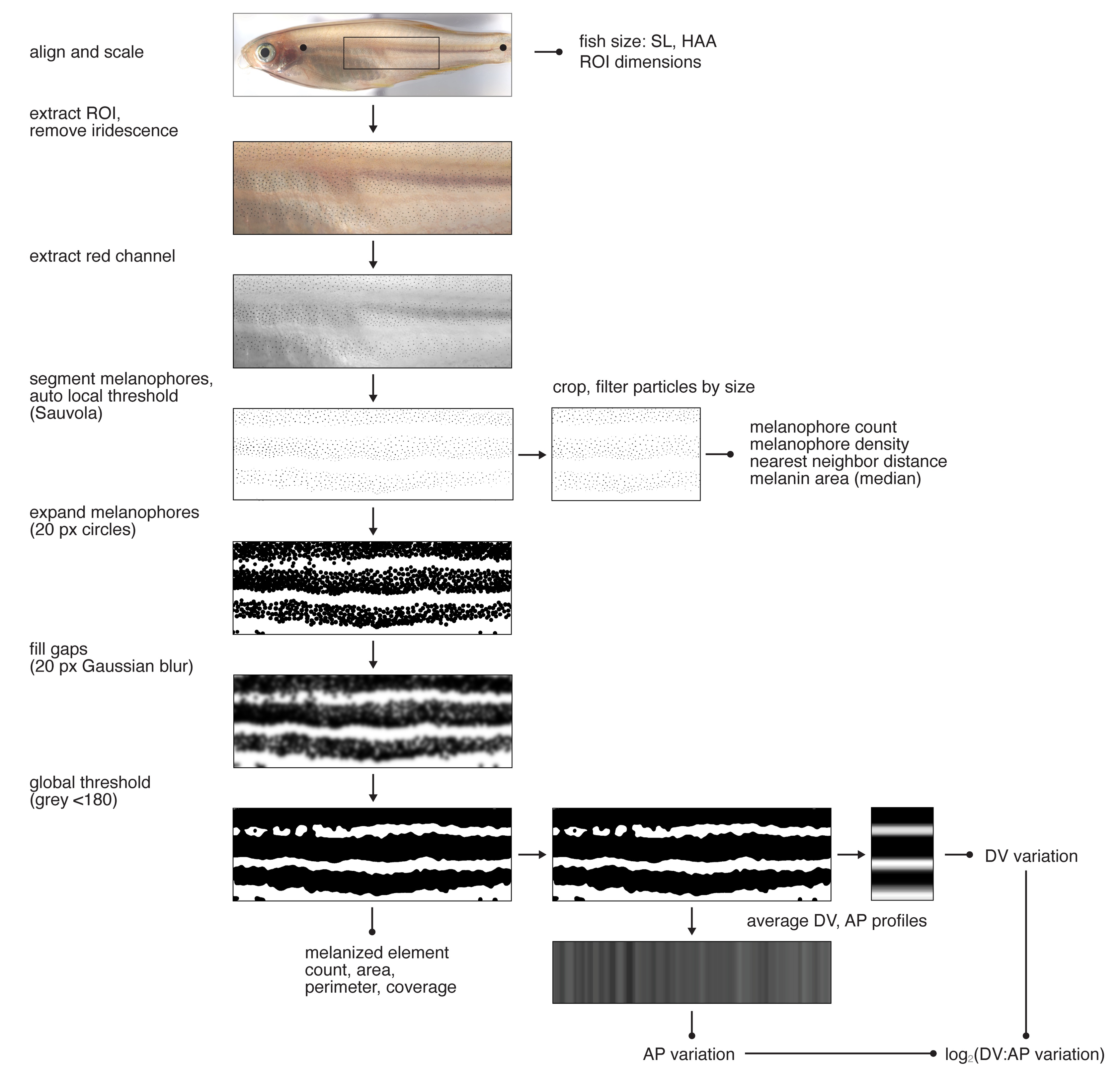

Supplement: S2 Fig — Whole-fish images were measured for size morphometrics, then isometrically scaled based on opercle and caudal peduncle and aligned so the primary interstripe was horizontal. The region of interest (ROI) was isolated and glare of iridophores removed followed by isolation of the red channel alone for visualizing melanophores in grey scale. Melanophores, defined by spots of contracted melanin pigment, were then segmented and thresholded. Additional cropping and filtering for size maxima and minima were applied for assessing cellular level metrics. The same approach was applied to xanthophores using gray scale images derived from the blue and red channels. For global pattern, melanized regions were expanded and then blurred to fill gaps, allowing for overlap similar to true cell edges (Fig 1 “natural”), and a global intensity threshold applied, defining melanized elements from which pattern metrics could be extracted directly (count, area, perimeter, relative coverage) or after isolating and averaging DV and AP grey value profiles. (TIF) [file pgen.1009364.s002.tif]

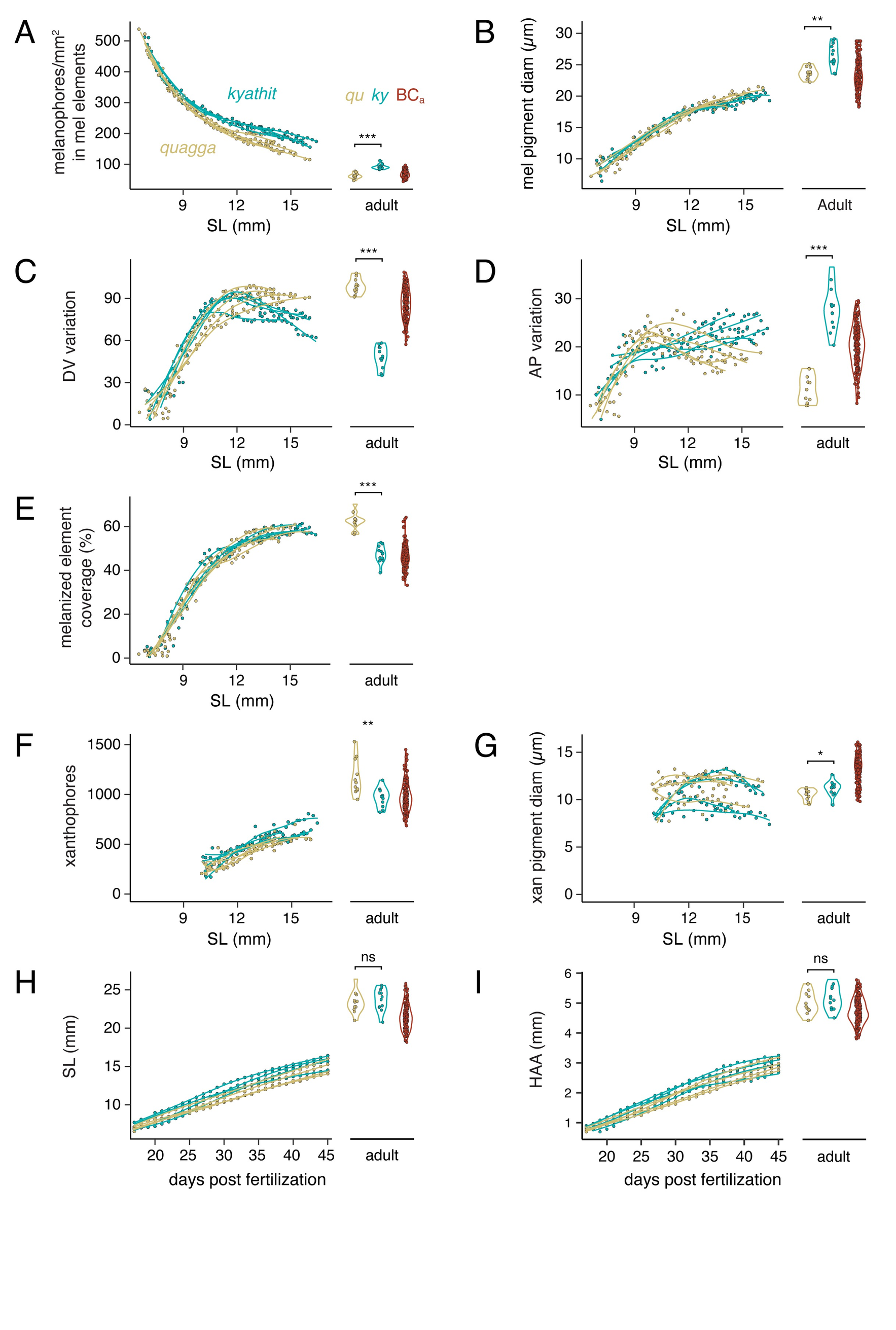

Supplement: S3 Fig — Additional quantitative metrics of patterns representing melanophore spacing and melanized pattern element distribution (A–E), xanthophore number and pigment (F,G), and body size (H,I). (A) Melanophore densities were greater in D. kyathit than D. quagga, though densities overall fell during development as fish grew in size. (B) Melanin content of individual melanophores did not differ during early adult pattern ontogeny but was significantly greater in older adult D. kyathit than D. quagga, as inferred from two-dimensional area of contracted melanin granules [54]. Values shown are medians for all melanophores quantified within an individual fish. (C) DV pattern variance continued to increase in both species as rudimentary primary stripes become increasingly organized and variation continued to increase in D. quagga as secondary stripes were added dorsally and ventrally. In D. kyathit, however, melanophores initially in primary stripes, as well as melanophores further dorsally and ventrally, clustered into spots, causing a progressive reduction in DV variation as some dorsoventral transects came to have little coverage by melanized pattern elements. (D) AP variation initially increased in both species, but then fell in D. quagga as anteroposteriorly oriented stripes became more orderly. AP variation increased in D. kyathit as initially continuous, rudimentary stripes were broken into spots, such that anteroposterior transects crossed spot–interspot boundaries. (E) The percent of the flank covered by melanized pattern elements was initially similar between species, though adults of D. quagga ultimately had greater coverage than D. kyathit. (F,G) Xanthophores first became visible later in development than melanophores and were ultimately more numerous in D. quagga than D. kyathit, though having slightly more visible pigment in the latter. (H,I) During development, D. kyathit were slightly larger than D. quagga (least squares means, SL: 11.8 vs. 11.1 mm, pooled SE = 0.0 [file pgen.1009364.s003.tif]

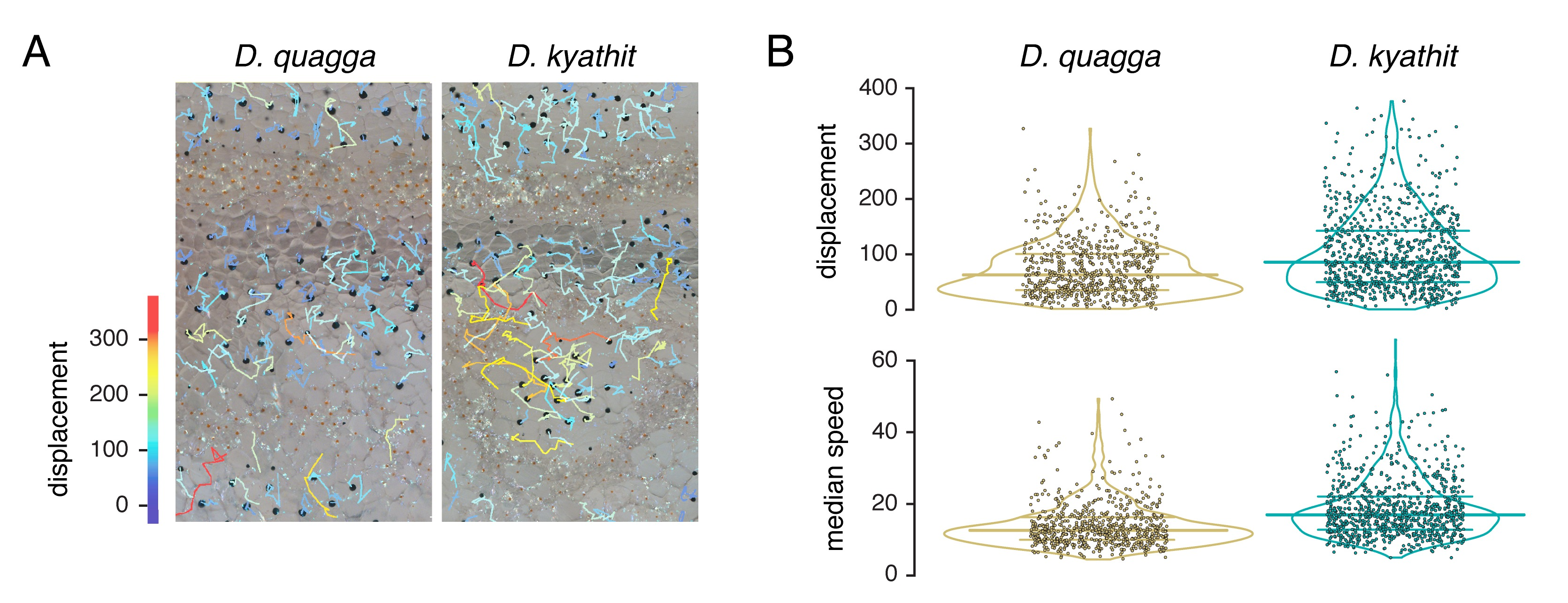

Supplement: S4 Fig — (A) Tracks indicate displacements of melanophores within regions shown in Fig 4A during the course of repeated imaging. (B) Total displacements and median speeds per day of melanophores observed during repeated imaging (pooled data for n = 4 larvae of each species). Horizontal bars indicate medians and interquartile ranges. As time points were isometrically scaled to allow for alignment and fish growth, all measurements are in scaled pixels. Analyses of variance for species differences, after controlling for nested (random) effects of individuals within species: displacement, F1,6 = 15.11, P = 0.0074; median speed, F1,6 = 7.15, P = 0.0366. Original values were ln-transformed and square root-transformed, respectively, to control for heteroscedasticity of residuals. (TIF) [file pgen.1009364.s004.tif]

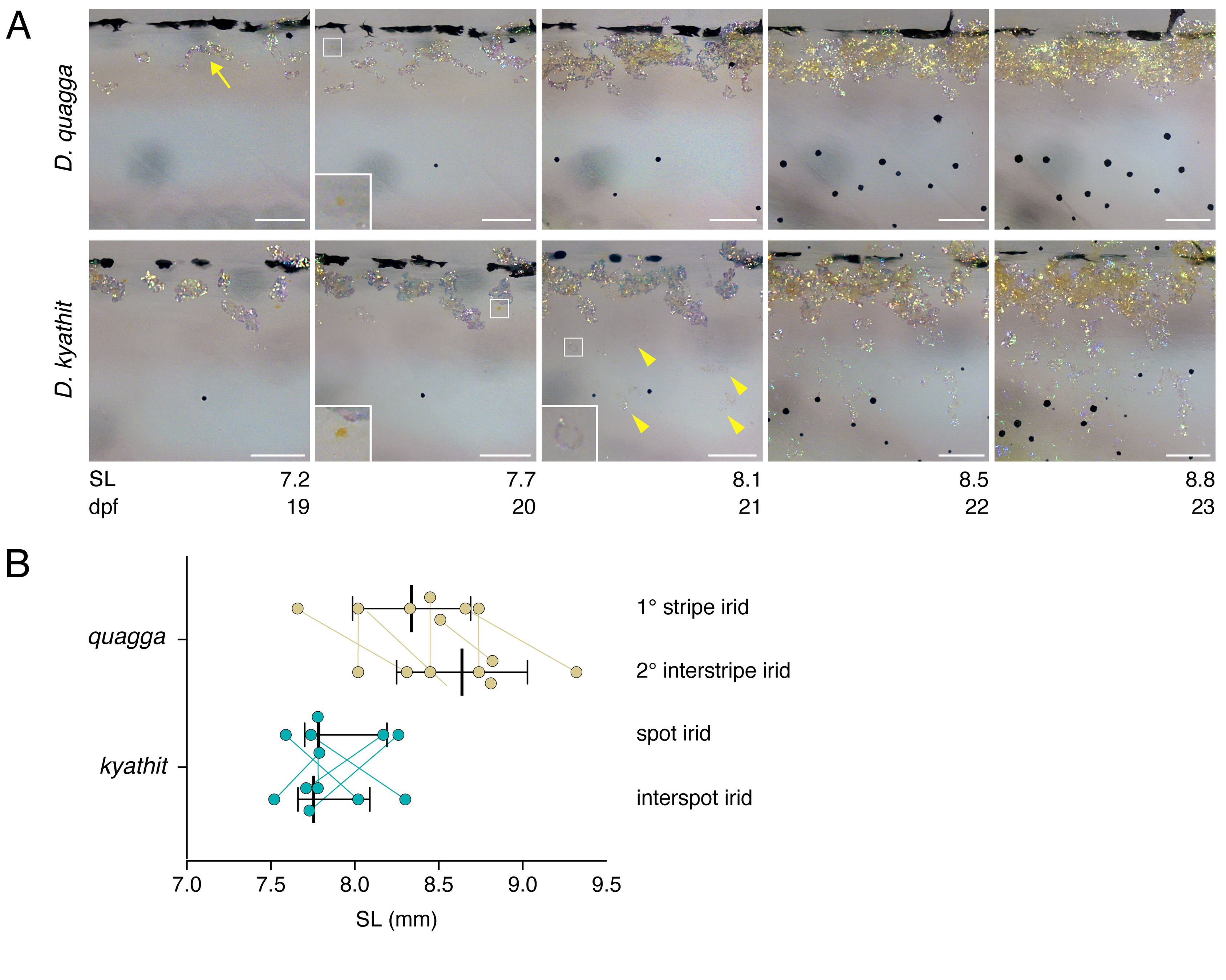

Supplement: S5 Fig — (A) After iridophores of the primary interstripe (e.g., arrow) developed, xanthophores began to differentiate nearby (insets, 7.7 mm SL), similar to D. rerio. Later, iridophores that will contribute to melanized pattern elements began to differentiate in D. kyathit (inset and arrowheads, 8.1 mm SL) and became more numerous thereafter, but iridophores had not yet appeared at these stages in D. quagga. (B) Sizes at which iridophores of stripes or spots or secondary, ventral interstripes or interspots were first evident in D. quagga (n = 7) and D. kyathit (n = 6) imaged daily through these stages of pigment pattern formation. Bars show medians with quartiles and observations from the same individuals are connected by lines. Species difference in multivariate analysis of variance, F1,11 = 22.64, P = 0.0007. Scale bars, 100 μm. (TIF) [file pgen.1009364.s005.tif]

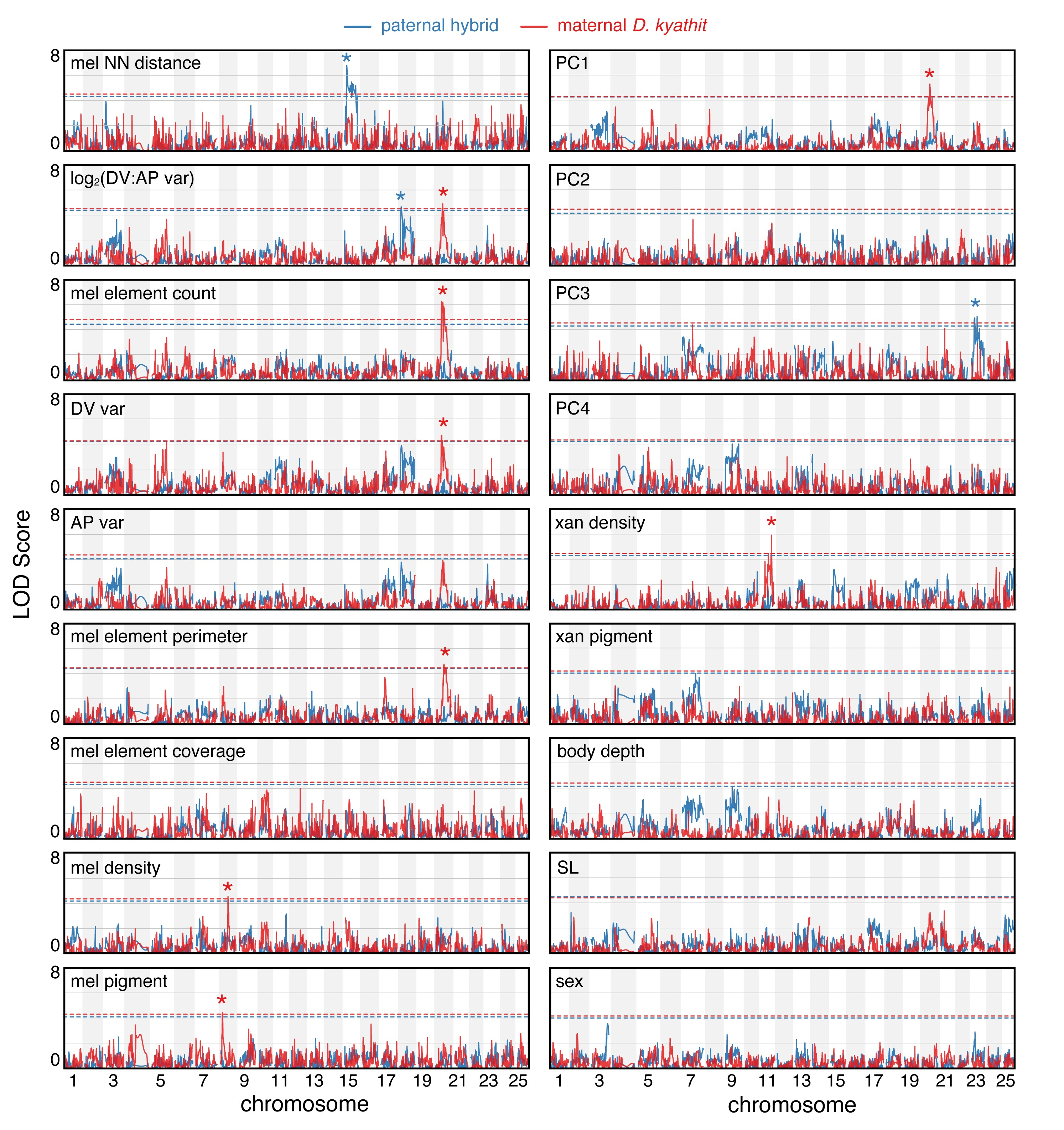

Supplement: S6 Fig — Chromosomal regions associated with example pattern and size metrics, sex, and principal components (PC) 1–4 in BCa progeny. Plots shown in Fig 6B–6D are shown here as well for ease of comparison. Asterisks indicate QTL exceeding 5% false discovery thresholds (dashed lines). (TIF) [file pgen.1009364.s006.tif]

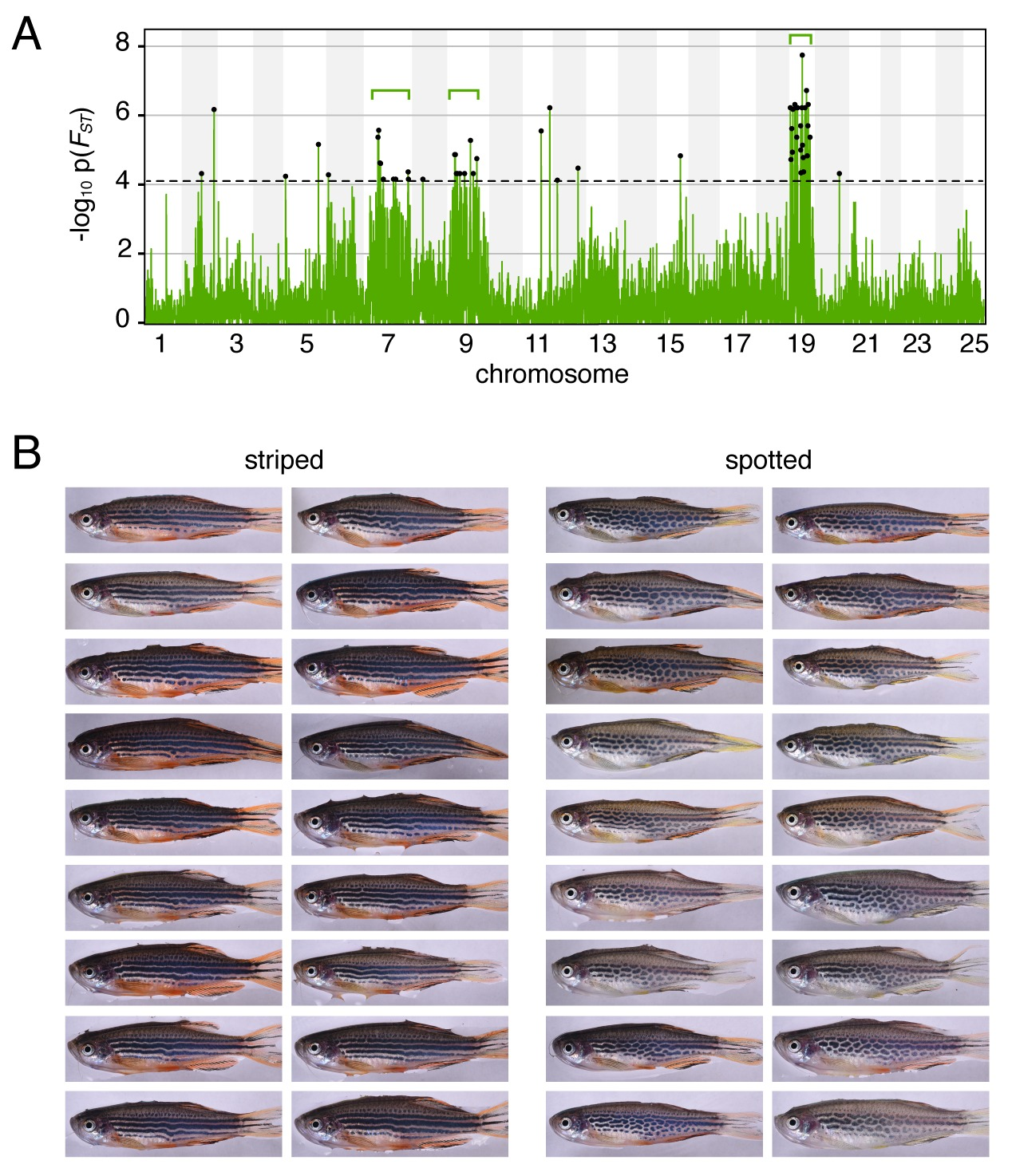

Supplement: S7 Fig — (A) Brackets indicate chromosomal regions with several loci having FST values exceeding an adjusted P-value of 0.01 (dashed line) and highlighted in Fig 6E. Isolated markers exceeding the threshold of statistical significance (e.g., Chr5) likely represent false-positives, because these analyses used full siblings and pattern-associated variants are physically linked to other variants; given the high marker densities achieved in these analyses, singleton peaks are more likely to reflect errors in mapping or genotyping. (B) Striped and spotted siblings used for FST scan. (TIF) [file pgen.1009364.s007.tif]

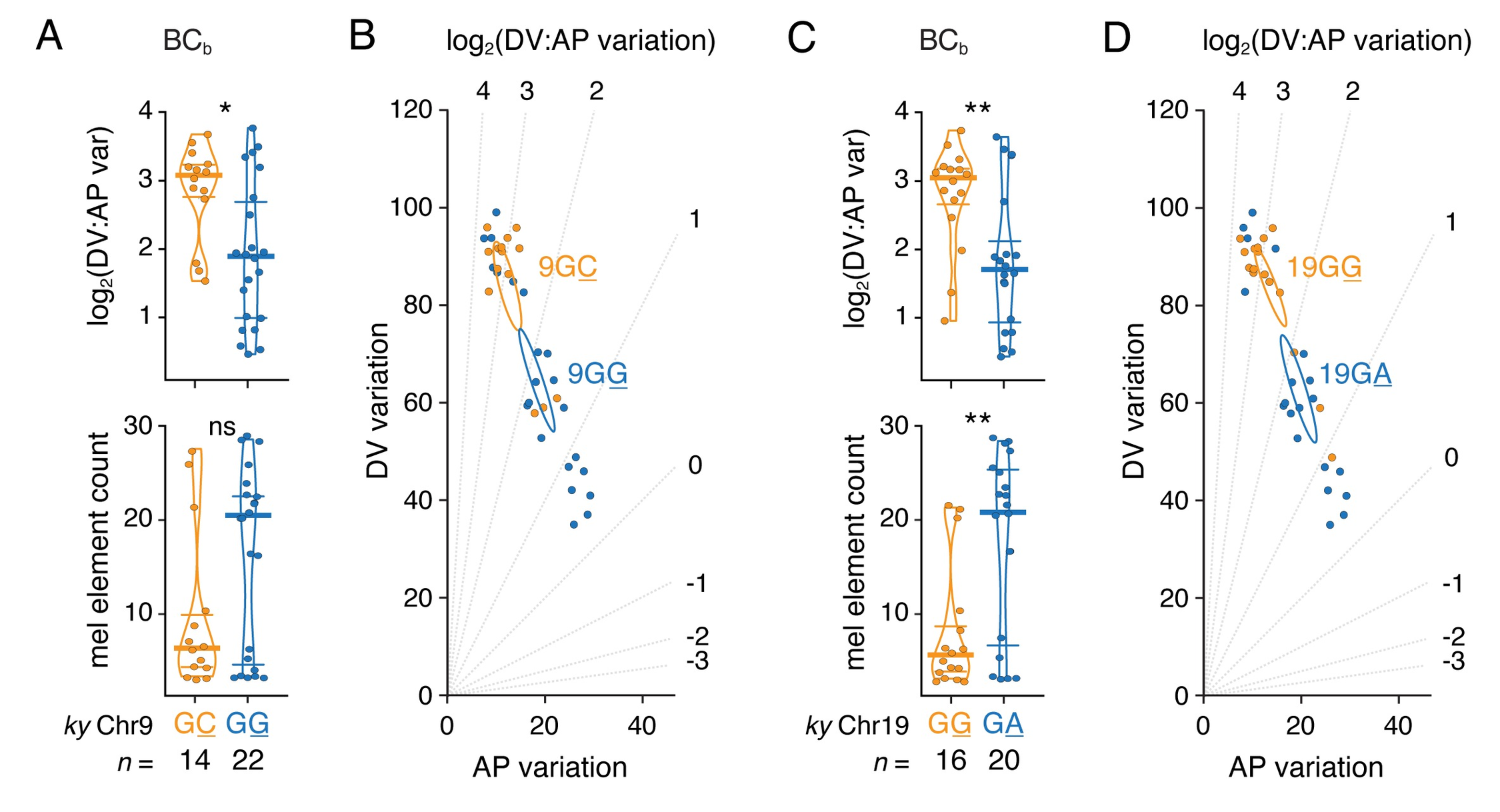

Supplement: S8 Fig — Panels show effects of alleles linked to variants at Chr9:36449721 (A,B) and Chr19:26824025 (C,D). Analyses of variance, in A: upper, F1,34 = 6.44, P = 0.0159; lower, F1,34 = 2.95, P = 0.0951; in C, upper, F1,34 = 7.57, P = 0.0094; lower, F1,34 = 8.47, P = 0.0063. Other annotations and analysis as in Fig 7. (TIF) [file pgen.1009364.s008.tif]
