# Supplementary material for: A complex genetic architecture in zebrafish relatives Danio quagga and D. kyathit underlies development of stripes and spots
Source: PLoS Genet. 2021 Apr 26;17(4):e1009364. doi: 10.1371/journal.pgen.1009364 (PMC8102007; doi:10.1371/journal.pgen.1009364)
Supplement: Attachment — Submitted filename: reply to reviewers.pdf [file pgen.1009364.s010.pdf]

# University of Virginia
The DEPARTMENT of BIOLOGY

March 28, 2021

Dear Robert and Greg.

We are very pleased by the overall positive responses to the manuscript and we thank the reviewers for their thorough assessment of the paper and suggestions for improvement. Below we address the reviewers comments; original comments are in black, our responses are in blue. Changes in the manuscript are marked in Track Changes.

Thanks for handling the manuscript,

Dave

---

This is a detailed, thorough and well-illustrated study, examining the genetic basis for naturally-evolved pigment patetrn differences in an experiemtnally accesible model system. Parichy and colleagues take advantage of phylogenetic proximity of two Danio sp. with distinct pigment patterns, and which form fertile hybrids, to begin to dissect the basis for natural pigment pattern evolution, in the context of the experimental studies in zebrafish which examine single locus mutations affecting coding regions and which generate large effects.

They begin by developing a quantitative measure of Danio pigment pattern, enabling ready comparison of spotted v striped patterns. Whilst in many ways this simply confirms the obvious visual differences seen in the 3 species, it has the advantage of giving an objective measure of the pattern differences, albeit restricted to melanophores, for both the DV and AP axes, and so comes into its own when examining rerio mutants and quagga x kyathit hybrids. The description in the Results of why they do this (p.4 Results second paragraph) is clear, but the description would benefit from slight expansion to explain the metrics used to define melanophore pattern; I am struggling to pin down what exactly the DV:AP ratio is measuring. The S3 Fig is helpful for explaining how images are processed, but doesn't quite explain this final step.

The averaged DV and AP profiles are one-dimensional arrays of gray values. We calculate the standard deviation of these arrays to determine DV and AP variation, then calculate the ratio of these two metrics to get $\log_2$(DV:AP variation). We modified the paragraph cited to include this this description, which will hopefully clarify the methodology and lead to a better intuition of the metric:

> "We therefore binarized patterns (melanophore element or not) and defined metrics to represent pattern phenotypes. These included estimates of pattern variation along dorsoventral (DV) and anteroposterior (AP) body axes, and the ratio between these metrics, most easily represented on a log scale, $\log_2$(DV:AP variation). To estimate variation along the DV body axis, we calculated the average grey value for each row (of individually black or white) pixels, representing a single anteroposterior transect across the body. From these average grey values across many rows (transects) in each individual, we then calculated standard deviations, and used these as our estimate of DV variation. We performed the reciprocal calculations to obtain for each individual an estimate of AP variation (i.e., standard deviation of averaged pix-

David M. Parichy Professor
Physical Life Sciences Building, 90 Geldard Drive, University of Virginia, Charlottesville VA 22903
dparichy@virginia.edu lab website

el values across DV column transects). Patterns or pattern elements that are nearly uniform along an axis will approach a value of 0, whereas increasing heterogeneity along an axis will yield increasingly large estimates of variation ”

Subsequently they perform a characteristically thorough and highly quantitative assessment of pigment pattern formation in both species. This section is very well documented, but somewhat overwhelming as presented. A summary table clarifying the observed similarities and distinctions in the evolution of pattern in the two species, with direct comparison to WT (and mutant?) zebrafish, would be helpful in highlighting clearly to the reader which features are candidates for being relevant to the switch between spots and stripes.

A summary table makes a lot sense, and we had ourselves been concerned with whether or not readers would get the “big picture” or would simply become bogged down in the details. We have added a qualitative summary as Table 1, to compare ontogenetic and terminal phenotypes of *D. kyathit* and *D. quagga*.

We think a full comparison of *kyathit* and *quagga* to zebrafish mutants is beyond the scope of this paper, which was intended to focus on natural genetic variation: our inclusion of wild-type and zebrafish nutants was intended primarily to validate the morphospace with additional phenotypes that will be familiar to some readers, and to illustrate that *kyathit* and *quagga* species differences are of similar magnitude to single locus phenotypes within zebrafish. An additional problem is that pattern ontogeny of many zebrafish mutants has not been assessed in a manner comparable to what we have done for *kyathit* and *quagga*, and so inferences would be tenuous without further data collection. In this regard, we are continuing our studies of *kyathit* and *quagga* with analyses that focus on specific pathways (e.g., gap junctional communcation) and we are using wild-type and zebrafish mutants in some of these analysses. So we anticipate that these kinds of comparisons will be included in a future study, likely in the next two or three years.

F1 hybrids between the 2 species were generated and patterns quantitated for comparison with parents, before backcrossing to kyathit for genetic characterisation by QTL mapping. Two independent backcrosses identify broad candidate regions on multiple chromosomes associated with a set of pigment pattern differences between the two species, although none were shared between the two backcrosses. None of these are directly characterised, but several are associated with known rerio pigmentation genes; however, the linked domains are very extensive, so it remains unclear if these known loci are contributing.

The authors main conclusion is that natural pigment pattern evolution in these two species appears to result from a complex genetic mechanism, involving multiple loci, similar to observations in other species.

Minor point:
p.11 ‘This situation contrasts with spotted mutants of D. rerio, in which melanophores are often fewer (e.g., ltk, gja5b, igsf11) [23, 38, 50] or of similar abundance (tjp1a, aqp3a) [51, 52] to that of wild-type.’ For this comparison will be useful, it seems that comparison of quagga and hyathit melanophore density to WT zebrafish is required.

Thanks for pointing this out. Our intended comparison was actually the nature of the difference between striped and spotted forms within each species context, as it was suprising to us that *kyathit* had more melanophores than *quagga*, when we had expected exactly the opposite. Though we have refrained from using our original language upon making this discovery (which might offend some readers), we modified our statement to make the point clearer:

“This situation implies that spots in *D. kyathit* are not simply a consequence of fish having insufficient numbers of melanophores to fill a striped pattern; this was surprising in comparison with spotted mutants of *D. rerio*, in which melanophores are often fewer (e.g., *ltk*, *gja5b*, *igsf11*) [23, 38, 50] or of similar abundance (*tjp1a*, *aqp3a*) [51, 52] to that of wild-type.”

---

Reviewer #1: Nicely conducted, and clearly reported, study. I have no significant concerns, as the data collection, and statistical analysis, have been carried out to a high standard. The general conclusion - that natural variation has a different, and more complex genetic basis than discovered in laboratory mutants - is not surprising to me, but is still not generally appreciated. This paper lays out a quantitative methodology that should be broadly applicable to other studies/systems.

Just a few minor typos remaining, for example:

Fig 6. Chromosomal regions associated with pattern and size metrics in F2 backross individuals. (backcross)

Fixed

---

Reviewer #2: In their manuscript "A complex genetic architecture in zebrafish relatives Danio quagga and D. kyathit underlies development of stripes and spots", McCluskey and co-authors provide an extremely thorough analysis of the genetic, cellular and developmental underpinnings interspecies variation in pigmentation pattern in Danio species. The authors first document the pigmentation phenotypes of the three Danio species, Danio rerio, Danio quagga (both striped) and Danio kyathit (spotted) at a cellular level. Next, they quantified dorso-ventral and anterior-posterior variation of melanic patterns across the Danio species complex and in mutant lines of the zebrafish, Danio rerio. This allowed the authors to compare the pattern variation by presenting the data in a 2-dimensional morphospace. The rest of the analyses focuses on the two sister species Danio quagga (striped) and Danio kyathit (spotted). A detailed analysis of pigment pattern development in the two species shows how the two phenotypes diverge during development. An analysis of the genetic basis using quantitative trait loci mapping provides insights into the (very polygenic) genetic basis of the phenotypic variation in pigmentation patterns between these two species. I very much liked this manuscript for three reasons:

i. The study investigates a phenotype (pigmentation patterns) from three completely different angles. It investigates the phenotypes from an evolutionary viewpoint by comparing pattern across species, provides a very detailed analysis of the developmental differences between two sister species that differ in their pigmentation and lastly and most importantly gives insights into the genetic basis underlying this variation. It therefore provides a uniquely complete and integrative analysis.
ii. The analyses conducted in this study have a very high quality and are very elaborate.
iii. I could imagine that it might be initially disappointing to realize that the identification of target genes (or even mutations) turned out to be so difficult because of the complex genetic basis. Nevertheless, I found the take-home message a very important one: That the genetic basis of natural variation is often much more complex, even if phenocopies could be generated by a single mutation.

I would like to congratulate the authors to their work. I have only a few, mostly minor suggestions.

Additional comments:

1) I initially thought that the F2 have been generated through inbreeding of F1 individuals. I think it would be helpful to indicate earlier that those are backcrosses. Also, a crossing scheme could be helpful, especially to more easily follow the results in Fig. 6D that are very complex. Also, the method/concept of QTL mapping could be maybe already introduced earlier (in the introduction; for example, in the paragraph "One powerful approach …" or in the last one).

The backcross origin of the studied individuals is now clarified with a crossing scheme added as Fig. 6A.

We additionally introduced the methodology of QTL mapping briefly in the penultimate sentence of the Introduction:

> "Finally, we utilize quantitative trait locus mapping to test, and reject, the hypothesis that a single major effect locus determines whether stripes or spots form, finding instead that multiple genomic regions contribute to pattern variation segregating in these laboratory crosses."

2) I enjoyed reading the introductory paragraph of the species. One information that would be nice to have there too, is the divergence time between Danio quagga and Danio kyathit and both to Danio rerio, also to know what "closely related" means in this fish family.

Estimating absolute divergence times among Cyprinid species is an ongoing struggle made particularly difficult by a lack of fossil evidence for small species like most *Danio* and an incredible diversity of mutation rates across taxa. The only molecular clock estimate for divergence time of *Danio* and related genera comes from a 2007 paper ("Evolution of miniaturization and the phylogenetic position of *Paedocypris*, comprising the world's smallest vertebrate," *BMC Evol Biol*), where Rüber and colleagues estimated the mitochondrial *cytb* nucleotide substitution rate within Cyprinidae as 0.0082 substitutions per site per million years. Yet they also showed the rate to be highly heterogeneous across taxa with danios and related species having the highest substitution rates. Estimating absolute divergence times using this best available estimate would, therefore, considerably overestimate divergence times between *Danio* species. A fuller study using a wider variety of cyprinid species (and danios in particular) would be the best way to determine an accurate estimate for absolute divergence time in this difficult family. At present, our best estimates of divergence are not absolute estimates in geological time, but rather estimates relative to other species in the genus, which helps explain the ability of these *quagga* and *kyathit* to form fertile hybrids where other species pairs generate sterile hybrids. Given all of these uncertainties, we have refrained from presenting specific estimates of divergence time, and we will leave it to readers to make their own inferences from the literature (e.g., discussion in McCluskey et al., *MBE 2015*).

3) Although the schematics are cute and helpful, I would consider adding actual photographs to Figure 2 on top. Also, seeing photographs of some of the Danio rerio mutants that are close to the D. kyathit morphospace would be a helpful visualization of one of the key messages (one could get there with a single mutation, too). Please ignore the request in case it is difficult because of copyright issues.

Figure 2 will follow soon after Figure 1, which contains whole-fish images of *kyathit*, *quagga*, *rerio*, so including images of those species in Figure 2 would be largely redundant.

We do not presently have comparable, publication quality images of all relevant species and cannot easily obtain new ones as some of our stocks had to be let go because of the pandemic. Inserting images piecemeal from other sources is problematic because of inconsistences in

lighting and resolution, and for copyright and other reasons. Consequently we have chosen to refer the reader to other sources in the legend of Figure 2:

> "Full color images of live fish can be found in [19] (open access); additional images can be found in [4, 16]."

4) While I found Figure 5 to be really interesting to understand the correlation between the cellular characteristics and pigmentation quantifications. This is now only a suggestion, but I kept wondering where the parental species and F1s would be in this PCA. If measurements for these are available a helpful visualization would be to perform a PCA with the parental species only and then project the F1s and BC individuals on the original PCA.

We appreciate the suggestion, but we have opted to maintain the current presentation for a number of reasons. First, using a PCA based on variation within $BC_a$ allows us to directly map the principal components of the variation segregating within that cross in Figures 6 and S7. Second, using principal components derived from the $BC_a$ individuals maximizes the loadings, allowing for clear labeling in this data-rich figure. Last, Figures 4 and S3 show the comparisons of individual pattern metrics between $BC_a$ and the parental species without conflating them onto a PCA space.

5) It would be great if the authors could expand a bit more on the method part of the QTL (how was the linkage map generated, how were markers filtered). Also, is there any evidence for larger rearrangements between these two species and the Danio rerio genome that could have affected the analysis (i.e., where linkage map and marker position on the D. rerio genome compared)?

We added to subsections "Genomic DNA extraction and genotyping" and "QTL mapping and statistical analyses" to expand on linkage map generation and marker filtering as requested:

> "Reads with multiple best mapping locations and reads mapping to annotated repetitive elements were excluded from further analyses."

> "Loci with any missing genotypes were excluded from analyses."

At present, there is no evidence of large rearrangements between zebrafish and more distantly related *Danio* species; translocations and large rearrangements seem unlikely to be at play between *quagga* and *kyathit* based on high survivorship of admixed progeny. It has long been known that a karyotype of 25 pairs of metacentric or submetacentric chromosomes is conserved across cyprinid species (except for polyploid species and scattered instances of chromosome fusions). Comparison of the zebrafish and common carp genomes in 2014 ("Genome sequence and genetic diversity of the common carp, *Cyprinus carpio,*" *Nature Genetics*) showed that intrachromosomal structure was also largely conserved between these divergent Cyprinid genera. As genome structure was highly conserved at the family level, we opted to map *quagga* and *kyathit* to the zebrafish reference genome and saw results consistent with karyotype conservation within *Danio*. If the QTL in this study spanned large inversions that occurred between these species, the associated loci in *quagga* and *kyathit* would map to multiple regions of a single chromosome when using the zebrafish genome as reference. Each of our QTL had a single peak, so we do not think this was a problem. The Sanger Institute has high quality, albeit still-unannotated genome assemblies of several *Danio* species (although *quagga* is not among them), and we (McCluskey, Postlethwait, Parichy) are collaborating with others to analyze and describe these genomes, which do not, so far reveal major rearrangements consistent with our inferences here and in the manuscript. Because we realize others may have similar concerns, we have added the following text to the Methods:

"Reads with multiple best mapping locations and reads mapping to annotated repetitive elements were excluded from further analyses. Mapping to the zebrafish genome allowed the use of existing annotations for inferring genes likely to be present within intervals defined by QTL. This approach is reasonable in the present context as the karyotype of 25 pairs of metacentric or submetacentric chromosomes is largely conserved across diploid species within Cyprinidae, which includes genus *Danio*, and comparison of zebrafish to common carp *Cyprinus carpio* revealed largely conserved intrachromosomal structure between even these divergent taxa [91-93]. Nevertheless, testing of individual candidates identified in this manner warrant further validation of candidate gene linkage with QTL as well as finer scale analysis of synteny relationships across these species."

6) In case genomic sequences of Danio quagga and Danio kyathit are available: Are there any coding differences between the target genes within the target intervals in Fig. 6 (tjpa, gja4, slc45a, kitla, …). I assume the responsible mutations are rather non-coding, but as comparing the coding sequences might be relatively straight-forward it might be worth checking.

We do not have access to genomes for the two species needed to perform these analyses. As many of the variants were present within *kyathit*, however, multiple genomes per species likely will be required to uncover the complex polygenic basis of the phenotypes in this study.

---

Reviewer #3: McCluskey and colleagues have submitted a manuscript describing an approach to quantify morphometric features for stripe/spot patterns in closely related danio fish species. They then used this quantification scheme to map QTL's of two closely related species of danio, one with stripes the other with spots that when intercrossed produced fertile offspring. They were able to determine regions on several chromosomes that had significant lod scores resulting in their being able to reject the single gene hypothesis for pattern differences. Further selective crosses identified other loci with variation both between species and within each species. Honestly the paper is well written, clear, the data of high quality. I have no significant critiques for improving the paper.

minor:
-fig 2 is not referenced in the text.

Actually the reference was near the end of the Introduction, where the phylogeny was mentioned just briefly. We also added a call-out in the Discussion where we reference the broader morphospace.

-I do wonder if the significant regions could have been narrowed rapidly and more finely by doing low coverage whole genome sequencing on pools of classified offspring looking for regions with loss of hetrozygosity. Not required for the paper, just curious.

As these fish are highly outbred, a loss of heterozygousity approach is not immediately applicable. Establishing inbred stocks for each species for such an approach may prove useful in the future, especially since many of the variants appear to be segregating within *kyathit*. But this approach has been difficult historically even for domesticated zebrafish stocks and we expect considerable hurdles owing to the exposure of recessive lethals, etc.

Reviewer #4: In this manuscript, McCluskey et al. make use of two closely-related Danio species with divergent pigment patterns to begin explore the genetics underlying these differences. This approach relies upon the ability to generate fertile hybrids of the two species, D. quagga and D kyathit, and a morphometric approach to quantify various elements of the adult pattern for the purpose of identifying QTL's. The study represents an alternative to the standard genetics of the Danio rerio model, by interrogating the nature of phenotypic differences in natural populations as opposed to those generated via laboratory-induced mutations.

The data in the paper are complex but presented with great clarity and rigor. Although the basic result that the stripe vs. spot difference between the two species is polygenic, and that while several genomic regions of interest are identified there are no "smoking guns" might be seen as slightly disappointing, in fact it validates the approach. This result is of great importance as biologists increasingly try to expand beyond just the traditional model systems.

My only suggestions:
Fig 5A presents shows a typical example of the F1 hybrid pattern, but I'd also be curious to see examples of the extremes in the backcross generation used for the Fst scan (perhaps added to S8 Fig). Also, are the individual male and female hybrids used for the backcrosses represented here? It would be interesting to know where each lies in the morphospace.

We added individuals used for the $F_{ST}$ scan to Figure S8 as suggested. The F1 individuals that were crossed to *kyathit* to generate the backcrosses were not plotted in morphospace.

If I understand correctly, the differences observed between the results of the two backcrosses (Figs 6 and 7) are likely due to interspecies variation as opposed to the sex of the parent-of-origin per se having some functional role? Perhaps that could just be clarified with a sentence somewhere.

Yes, we addressed the parent-of-origin issue by adding this sentence in the Results:

"To account for any parent-of-origin effects, we used a hybrid female in $BC_b$ as opposed to the hybrid male used in $BC_a$."

Small points:
p. 5 fourth line from the bottom: word "adult" is repeated

p. 6 last line before "Genetics of pigment pattern variation" subheading: "each having more specific effects"

S1 Fig legend third line from bottom: presumably should be "~3.5 cm SL", not 3.5 mm

Thanks. All fixed.

---

Reviewer #5: The manuscript by McCluskey et al. describes their approach to identify genetic bases determining 'stripes' or 'spots' adult pigment pattern in Danio species. Their study is very intriguing and challenging: they used two close relatives of Danio species which can produce fertile F1 progeny, thus enabling them to use F2 for mapping natural genetic variants between the two species, responsible for the different pigment patterns of 'stripes' or 'spots'. Unfortunately, their genetic analyses haven't reached a clear conclusion: instead of finding a simple main

effect locus involved, they revealed a complex genetic basis with several loci contributing to pattern variation. In overall, the results are convincing and the strategies described are valuable for future research by others.

Comments:
Their genetic analyses pointed out some loci, where the candidate genes known to be involved in pigment pattern formation are located. Are any of these responsible for the formation of stripe or spot?

As the two backcrosses in this study identified distinct QTL, we know that the genetic basis for pattern divergence between these species is polygenic. Determining which combination of these candidate genes or other genes in the numerous QTL are responsible for the pattern difference between *quagga* and *kyathit* will be very laborious and time-consuming and so we have had to keep that goal outside the scope of the present study, if only for the sake of grant renewals, career transitions, etc. Nevertheless, we hope to answer this question in the future with additional analyses that have been initiated already.

They don't mention about the difference across species in sequence there, nor in their expression (special, temporal or level). Are there any nucleotide alterations? If any, are they all non-coding?

A survey of variation in *quagga* and *kyathit* across the many current candidates, and potentially additional candidates is impractical without assembled and annotated genomes for both species. We expect numerous coding sequence changes exist within and between these species given what we know from surveys of zebrafish natural variation. For example, even within our smallest QTL (~10 Mb) containing a good candidate gene (*kita*), there are 108 annotated missense mutations among domesticated zebrafish strains, including two within the pigment-associated gene *kita*. Sorting out this kind of variation in natural populations, and assessing the functional impacts of such substitutions will not be trivial and will really require a dedicated study. We do not have spatiotemporal expression data for candidate genes across *quagga* and *kyathit*. Such data could be useful in future studies, but will likely require the application of scRNA-Seq approaches, given heterogeneities in pigment cell states through development. Though we have done this, and continue to do this for zebrafish, such analyses require genomic resources not yet available for *quagga* and *kyathit*.

Their imaging analyses for chromatophore behavior upon pigment pattern formation in the two species revealed different timing of melanophore differentiation is key to generate distinct pattern, 'stripes or spots', suggesting that heterochrony should explain for this interspecies difference. Are the above loci related to the heterochrony?

Previous work from my group ("Evolution of Endothelin signaling and diversification of adult pigment pattern in *Danio* fishes," *PLoS Genetics* 2018) revealed an alteration in Endothelin-3b expression (which falls within the Chr23 QTL in the present study) that led to a heterochrony in pigment pattern development between zebrafish and *D. nigrofasciatus*. We documented other heterochronies, in Csf1 expression and xanthophore development, between zebrafish and D. albolineatus ("Pigment cell interactions and differential xanthophore recruitment underlying zebrafish stripe reiteration and *Danio* pattern evolution," *Nature Communications* 2014). It is possible, and maybe even likely, that heterochronic differentiation of pigment cells (specifically iridophores) is the distinguishing feature of the pigment pattern differences between *quagga* and *kyathit*, though it also could also be a correlated effect with a separate cause. Our own feeling is

that heterochonic shifts are pervasive in generating species differences, but in 2021 we think they are best discussed and interpreted in the context of molecular and cellular mechanisms. So we are very enthusiastic about the reviewer's suggestion and we hope to address the issue more comprehensively in future work.

'Morphospace' part is hard to understand, especially Fig 5B. Where is Fig 5C?

The data presented in Figure 5 includes both the metrics we used to define DV and AP morphospace and additional descriptors of the pattern differences among backcross progeny. So it really is intended to be more comprehensive than the morphospace described in Fig 3. We agree that plots of principal components can be somewhat difficult to interpret, but we have not been able to find a satisfactory alternative representation without adding substanially to the manuscipr length and figure content. Regarding the Fig 5C call-out, this was a relic from a previous iteration of the figure and so has been removed from the manuscript.

In Fig. 7, is A consistent with B in genotypes? Is the maternal allele always indicated first (left)? Why are the genotypes different between 7A and 7B?

Both Fig 7A and Fig 7B show the same genotypes. The maternal allele is always indicated first in Fig 7B, but not in Fig7A. We have adjusted the labels in Fig 7A to maintain this convention (i.e., 18AG was changed to 18GA).

SL appears prior to spelling-out (page 6, 2nd and 3rd paragraph). Duplicate 'adult' (page 5, last paragraph).

Thanks. We changed SL to "standard length" and removed the duplication.

---

Have all data underlying the figures and results presented in the manuscript been provided? Large-scale datasets should be made available via a public repository as described in the PLOS Genetics data availability policy, and numerical data that underlies graphs or summary statistics should be provided in spreadsheet form as supporting information.

Reviewer #1: Yes

Reviewer #2: No: No, but the authors have declared that data will be available upon publication through public repositories.

Reviewer #3: Yes

Reviewer #4: Yes

Reviewer #5: Yes

Illumina sequence data has been made publicly available through the short read archive, and accession PRJNA691921 has been added to the title page. All numerical data underlying graphs is provided in spreadsheet form as Supporting Information. Files used for identifying and mapping QTL through the QTL2 package in R have been uploaded to the Zenodo Digital Repository (https://zenodo.org/record/4638220).
